# Formation of near-vertical trenches and fins with flat {−201} sidewalls on (512) β-Ga_2_O_3_ via TMAH wet etching

**DOI:** 10.1080/14686996.2026.2726186

**Published:** 2026-09-11

**Authors:** Takayoshi Oshima

**Affiliations:** Research Center for Electronic and Optical Materials, National Institute for Materials Science, Tsukuba, Japan

**Keywords:** β-Ga_2_O_3_, (512), wet etching

## Abstract

We achieved the formation of near-vertical trenches and fins with exceptionally flat {−201}-faceted sidewalls on (512) β-Ga_2_O_3_ substrates by crystallographic wet etching in 25-wt% tetramethylammonium hydroxide (TMAH). Since wet-etched {−201} surfaces exhibit atomically flat step-and-terrace morphologies, substrate orientations perpendicular to the {−201} plane and having high wet-etch rates are expected to enable smooth vertical trench/fin formation by wet etching alone. To identify such orientations, we measured the in-plane dependence of the side-etch rate on a (−201) substrate and found that side etching was most enhanced near the (512) and equivalent planes. We therefore investigated the wet-etch characteristics of (512) substrates. The planar etch rate increased exponentially with temperature, reaching 0.724 μm h^−1^ at 90°C, indicating surface-reaction-limited behavior with an activation energy of 83.3 kJ mol^−1^ (0.863 eV). Near-vertical etching was achieved only for etching windows aligned along the [1−92] direction, parallel to the intersection between the (−201) and (512) planes. The developed {−201}-faceted sidewalls were flat and slightly inclined from the (512) surface normal by approximately 1.8°, in good agreement with the crystallographically estimated angle of 1.70°, suggesting that perfectly vertical etching could be achieved using 1.70°-miscut (512) substrates.

## IMPACT STATEMENT

This study first demonstrates that (512) β-Ga_2_O_3_ enables the formation of near-vertical trench and fin structures with exceptionally flat {−201} sidewalls by wet etching alone.

## GRAPHICAL ABSTRACT

**Figure:**
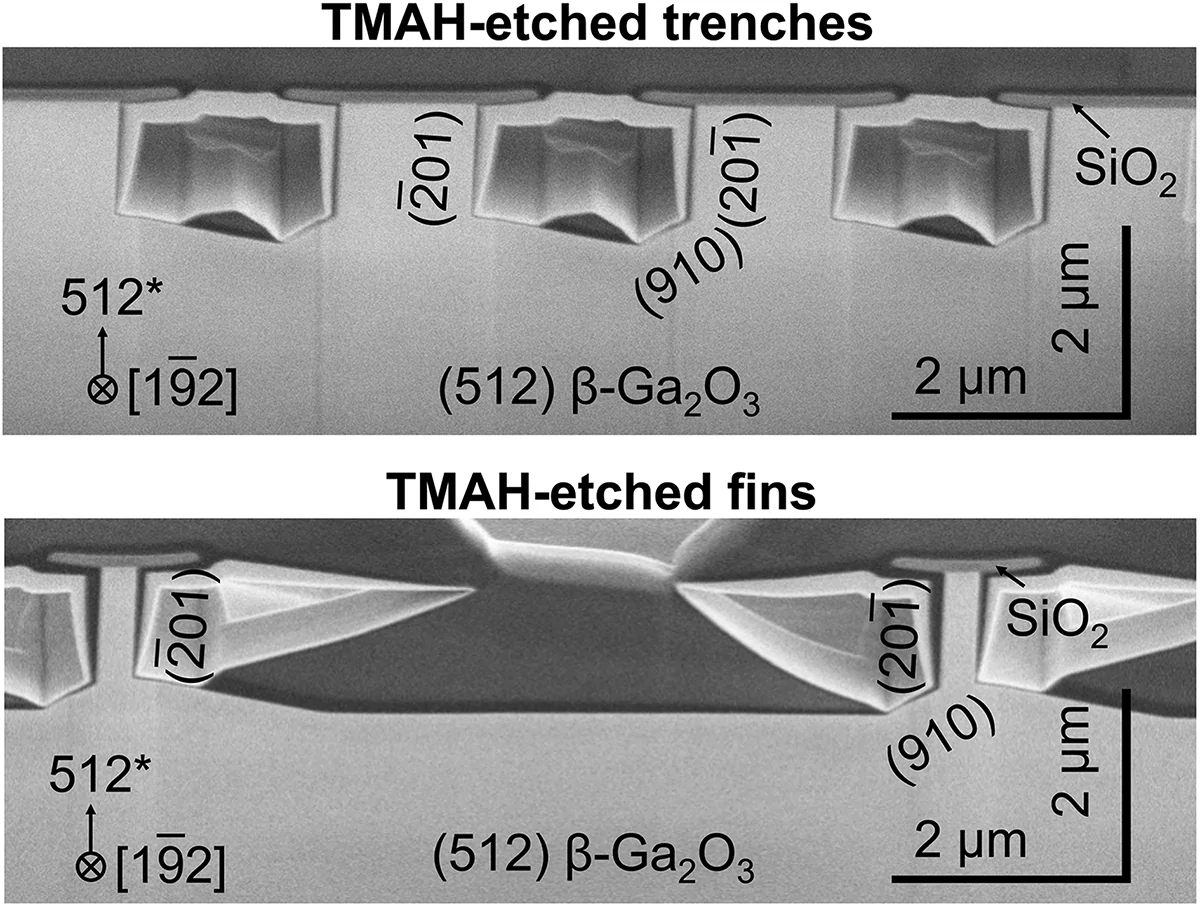

## Introduction

β-Ga_2_O_3_ has recently attracted considerable attention as an ultra-wide-bandgap semiconductor and is regarded, alongside SiC, GaN, and diamond, as one of the key power semiconductors capable of overcoming the material limits of Si [1,2]. Moreover, β-Ga_2_O_3_ offers the advantage of melt-growth-based bulk crystal production [3] and compatibility with a wide range of processing technologies commonly used in semiconductor fabrication, including epitaxial growth [4], ion implantation [5], dry and wet etching [6], ion cutting and wafer bonding [7]. Such exceptional process flexibility has accelerated the research and development of β-Ga_2_O_3_-based power devices, contributing to their rapid progress relative to other wide- and ultra-wide-bandgap power semiconductors [1,2].

Among these processing technologies, we focus on crystallographic wet etching. Crystallographic wet etching is an anisotropic etching technique that exploits orientation-dependent etch rates to produce well-defined microstructures bounded by specific crystal planes. This technology is well established for Si, where highly etch-resistant {111} facets are preferentially exposed during wet etching, enabling the formation of various structures—such as inverted pyramidal pits, V-shaped grooves, and vertical trenches and fins—through appropriate selection of the substrate orientation and mask pattern [8]. In particular, crystallographic wet etching is a key Si processing technology for fabricating microelectromechanical systems (MEMS) [8].

In contrast, wet etch patterning of β-Ga_2_O_3_ has been reported only in a limited number of studies [9–18], and crystallographic wet etching for the intentional formation of specific faceted structures has been demonstrated in even fewer works [15,16,18]. In crystallographic wet etching of β-Ga_2_O_3_, {$\overset{ˉ}{\text{2}}$01} and {001} facets tend to be exposed, resulting in exceptionally flat {$\overset{ˉ}{\text{2}}$01} and relatively flat {001} etched surfaces [15,16]. In our previous studies, we utilized this etching behavior to form step-and-terrace surface morphologies with (001) terraces on (001) substrates [16], V-shaped grooves consisting of ($\overset{ˉ}{2}$01) and (001) facets along the [010] direction on ($\overset{ˉ}{1}$02) substrates [18], and vertical trenches with {$\overset{ˉ}{2}$01} and {001} sidewalls along the [102] and [100] directions, respectively, on (010) substrates [15]. Among these structures, vertical trenches and fins are particularly important for power electronics applications, such as trench Schottky barrier diodes [19] and fin field-effect transistors [20]. However, owing to the low etch rate of the (010) surface [10,15,17], such trench and fin structures had to be initially defined by dry etching prior to the crystallographic wet etching process on (010) substrates [15]. Therefore, the formation of vertical trenches and fins by wet etching alone requires the identification of an appropriate substrate orientation.

In this study, we explored substrate orientations suitable for forming vertical trenches and fins with flat {$\overset{ˉ}{2}$01} sidewalls solely by wet etching, and identified slightly misoriented {512} planes as such orientations for this purpose.

## Experimental methods

We conducted wet etching experiments on Sn-doped ($\overset{ˉ}{2}$01)- and (512)-oriented β-Ga_2_O_3_ substrates covered with patterned SiO_2_ masks in heated tetramethylammonium hydroxide (TMAH). The substrate orientations were confirmed by the substrate manufacturer (Novel Crystal Technology) using X-ray diffraction measurements. According to the specification sheets, the nominal miscut angles of the (512) substrate were −0.2° toward ≈[20$\overset{ˉ}{5}$] (the direction perpendicular to [$\overset{ˉ}{1}9\overset{ˉ}{2}$]) and 0.0° toward [$\overset{ˉ}{1}9\overset{ˉ}{2}$], while those of the ($\overset{ˉ}{2}$01) substrate were 0.2° toward [010] and −0.7° toward [102]. The SiO_2_ masks were approximately 0.1 μm thick and contained various window patterns. They were prepared by plasma-enhanced chemical vapor deposition, laser lithography, and subsequent etching using either buffered HF or capacitively coupled plasma reactive ion etching. Wet etching was performed in a polytetrafluoroethylene (PTFE) container with a closable lid, as schematically illustrated in our previous paper [15]. The process temperature was controlled using a perfluoroalkoxy-alkane-coated thermocouple immersed in 25-wt% TMAH, and the solution was stirred with a PTFE-coated stirring bar to ensure temperature uniformity.

The processed samples were characterized as follows. The wet-etched surface morphologies were examined using atomic force microscopy (AFM). The planar etch rates were measured using a surface profilometer. The side-etch lengths were determined from optical microscopy and scanning electron microscopy (SEM) images by measuring the distance between the mask edge and the etching front beneath the mask. SEM was also used to observe etched structures, including cross-sectional profiles. The cross sections were exposed by focused ion beam milling after local deposition of a carbon protective layer to preserve the surface profiles. The SEM acceleration voltage was set to 2 or 10 kV, depending on the observation purpose: 2 kV was used to observe fine structures with enhanced material contrast, whereas 10 kV was used to visualize side-etched structures beneath the mask [21].

## Results and discussion

**Figure 1.**
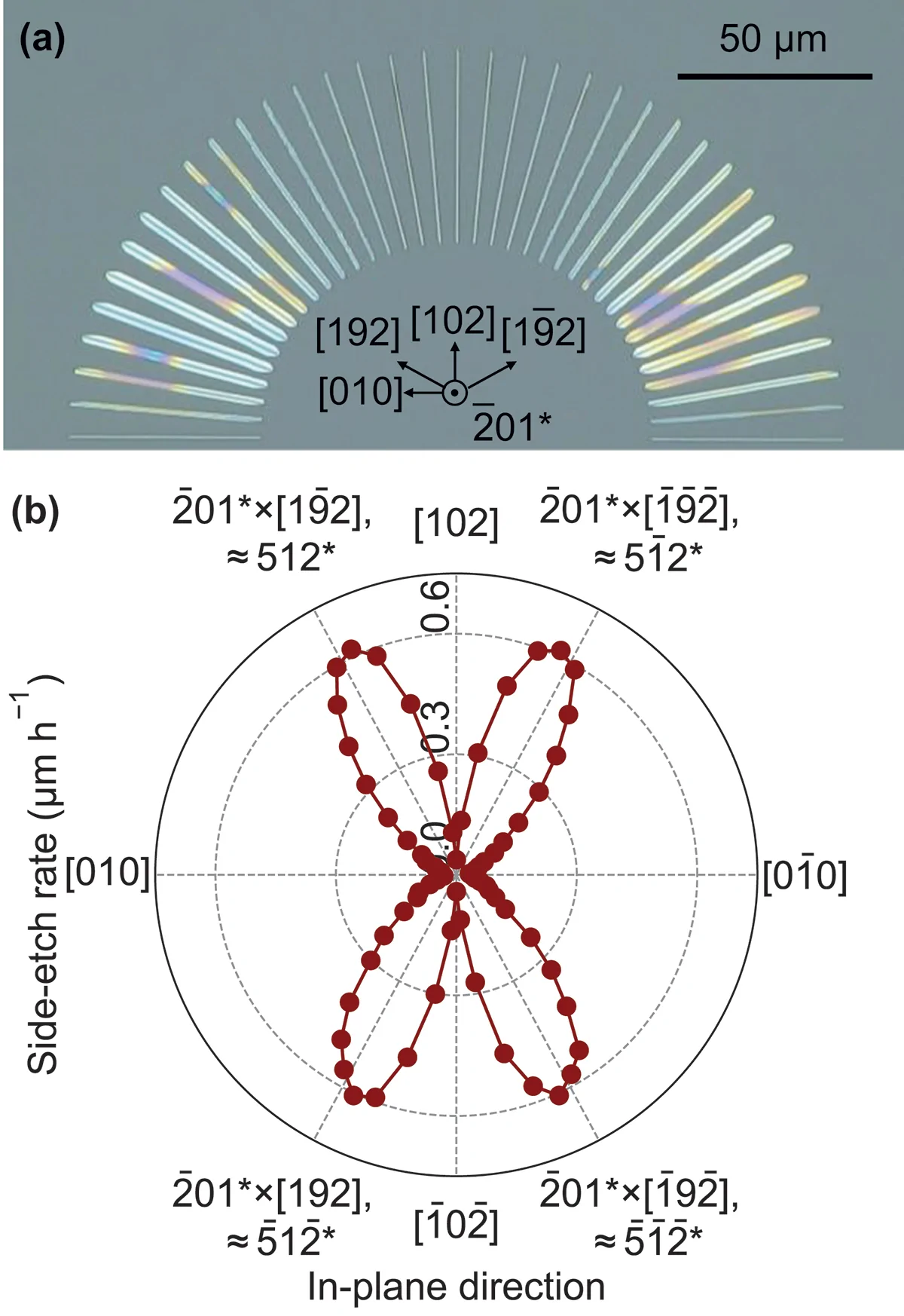
(a) Optical microscopy image of wet-etched trenches formed beneath wagon-wheel windows on a ($\overset{ˉ}{2}$01) β-Ga_2_O_3_ substrate. The lower-half windows are not shown, as they do not provide additional information beyond that obtained from the upper-half windows. (b) Polar plot of the side-etch rate derived from the wagon-wheel-patterned trenches. Here, ‘*’ and ‘×’ denote the reciprocal lattice vector and cross product, respectively. ‘≈’ indicates that the direction of the resulting vector is approximately parallel to the corresponding plane normal, with a deviation of approximately 1.7°; only the direction of the vector, not its magnitude, is considered.

We first evaluated the side-etch characteristics on the ($\overset{ˉ}{2}$01) β-Ga_2_O_3_ substrate to find in-plane orientations exhibiting high etch rates, as shown in Figure 1. For the evaluation, we used wagon-wheel-patterned linear windows with a width of 0.74 μm and a length of 50.3 μm arranged at 5° intervals from the [0$\overset{ˉ}{1}$0] direction. The TMAH etching was performed at 90 °C for 2 h. The etched trenches formed beneath the windows exhibited anisotropic side-etch behavior, as shown in an optical microscopy image (Figure 1(a)), where the side-etched regions were clearly distinguished by contrast from the mask and window regions. Side etching was enhanced for windows aligned along the [192] and [1$\overset{ˉ}{9}$2] directions, which are crystallographically equivalent and are parallel to the intersections between the ($\overset{ˉ}{2}$01) and the {512} planes—namely, (512), (5$\overset{ˉ}{1}$2), ($\overset{ˉ}{512}$), and ($\overset{ˉ}{5}$1$\overset{ˉ}{2}$). The side-etch length, defined as the distance between the mask edge and the etching front, was measured to extract the side-etch rate, which is summarized in polar coordinates in Figure 1(b). The resulting side-etch-rate profile showed four maxima along in-plane directions that are perpendicular to the [192] and [1$\overset{ˉ}{9}$2] directions and close to the {512} surface normals, suggesting relatively high etch rates for the {512} planes. Note that the {512} planes are nearly perpendicular to the ($\overset{ˉ}{2}$01) plane; the (512), (5$\overset{ˉ}{1}$2), ($\overset{ˉ}{512}$), and ($\overset{ˉ}{5}$1$\overset{ˉ}{2}$) planes are tilted by 1.70° from the ($\overset{ˉ}{2}$01) surface normal, respectively. This nearly perpendicular relationship can also be understood in terms of the pseudo-cubic oxygen sublattice of β-Ga_2_O_3_ [22]. In this framework, the {512} and {$\overset{ˉ}{2}$01} planes correspond to the {110} and {111} planes of a face-centered-cubic (fcc) oxygen sublattice, respectively. Specifically, the (512) and ($\overset{ˉ}{2}$01) planes in β-Ga_2_O_3_ correspond to the (101) and (11$\overset{ˉ}{1}$) planes, respectively, and these two fcc planes are perpendicular in an ideal fcc lattice.

**Figure 2.**
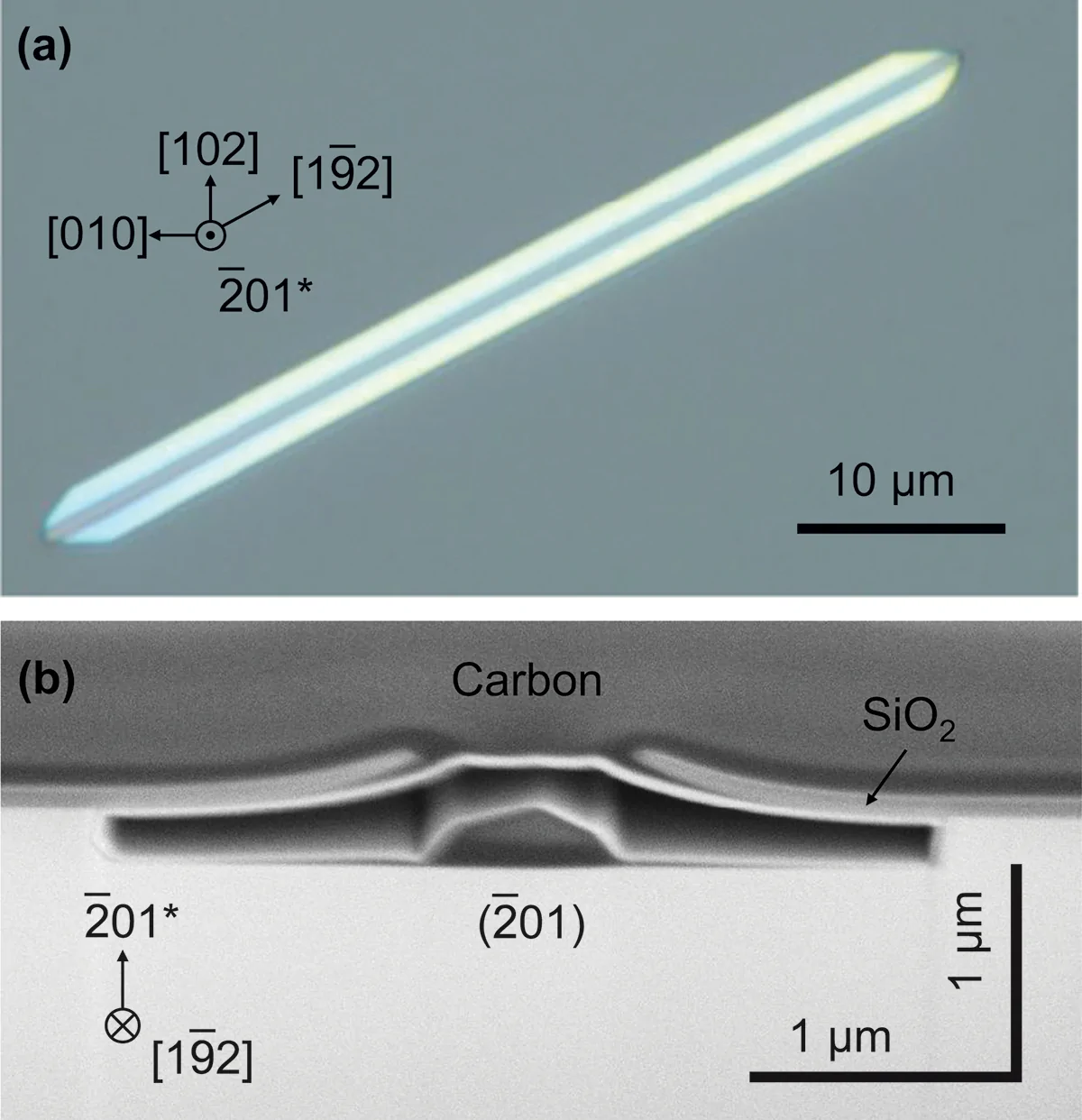
(a) Optical microscopy image of a wet-etched trench aligned along the [1$\overset{ˉ}{9}$2] direction. (b) 54°-tilted-view cross-sectional scanning electron microscopy image of the wet-etched trench shown in (a). Note that the vertical and horizontal scales on the cross section are different due to the tilted view.

**Figure 3.**
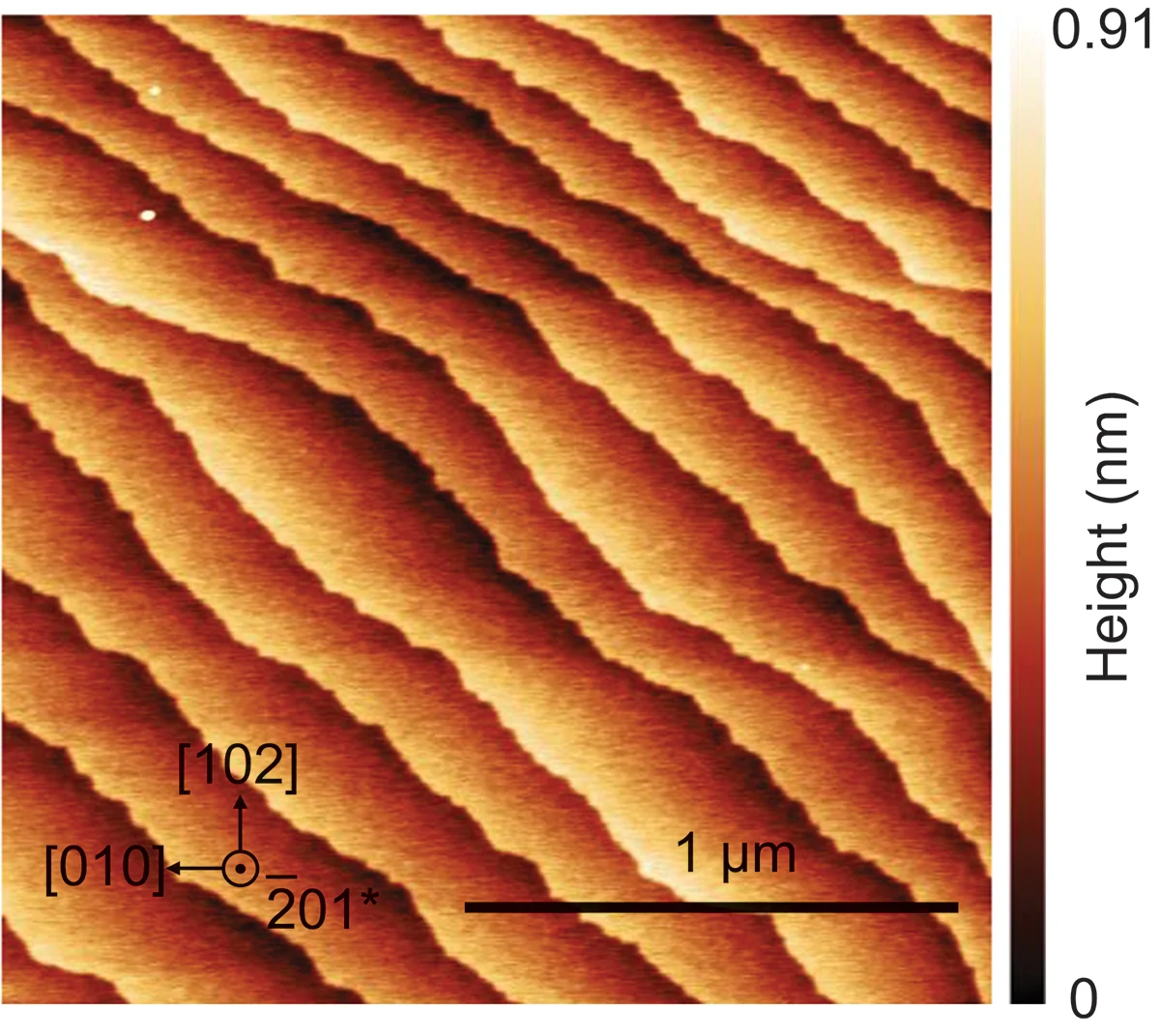
Surface morphology of the wet-etched ($\overset{ˉ}{2}$01) substrate observed by atomic force microscopy. The root-mean-square roughness was 0.15 nm.

We further examined the cross-sectional profile of the etched trench beneath a linear window aligned along the [1$\overset{ˉ}{9}$2] direction on the same ($\overset{ˉ}{2}$01) substrate, as shown in Figure 2. The window width and length were identical to those of the wagon-wheel windows. Figure 2(a) shows an optical microscopy image of the trench, in which the side-etched regions were also clearly visible. Figure 2(b) shows a cross-sectional SEM image of the trench, where the cross section was perpendicular to the [1$\overset{ˉ}{9}$2] direction. The cross-sectional profile revealed laterally enhanced etching, with lateral etch rates of ≈0.59 μm h^−1^, which were about 5 times higher than the vertical etch rate ≈0.12 μm h^−1^. Furthermore, the etched bottom surface was flat and horizontal even in the side-etched regions, indicating that the ($\overset{ˉ}{2}$01) surface was maintained. Note that the etched ($\overset{ˉ}{2}$01) surface exhibited step-and-terrace morphology with a very small root-mean-square (RMS) roughness of 0.15 nm, as evidenced by the AFM image acquired over a 2 μm × 2 μm scan area within a 200 μm × 200 μm window opening, as shown in Figure 3. These results suggest that nearly vertical wet etching with a reasonable etch rate and flat sidewalls may be achievable by selecting {512} substrate orientations. Therefore, we procured custom-fabricated (512) β-Ga_2_O_3_ substrates and investigated their wet-etch characteristics.

**Figure 4.**
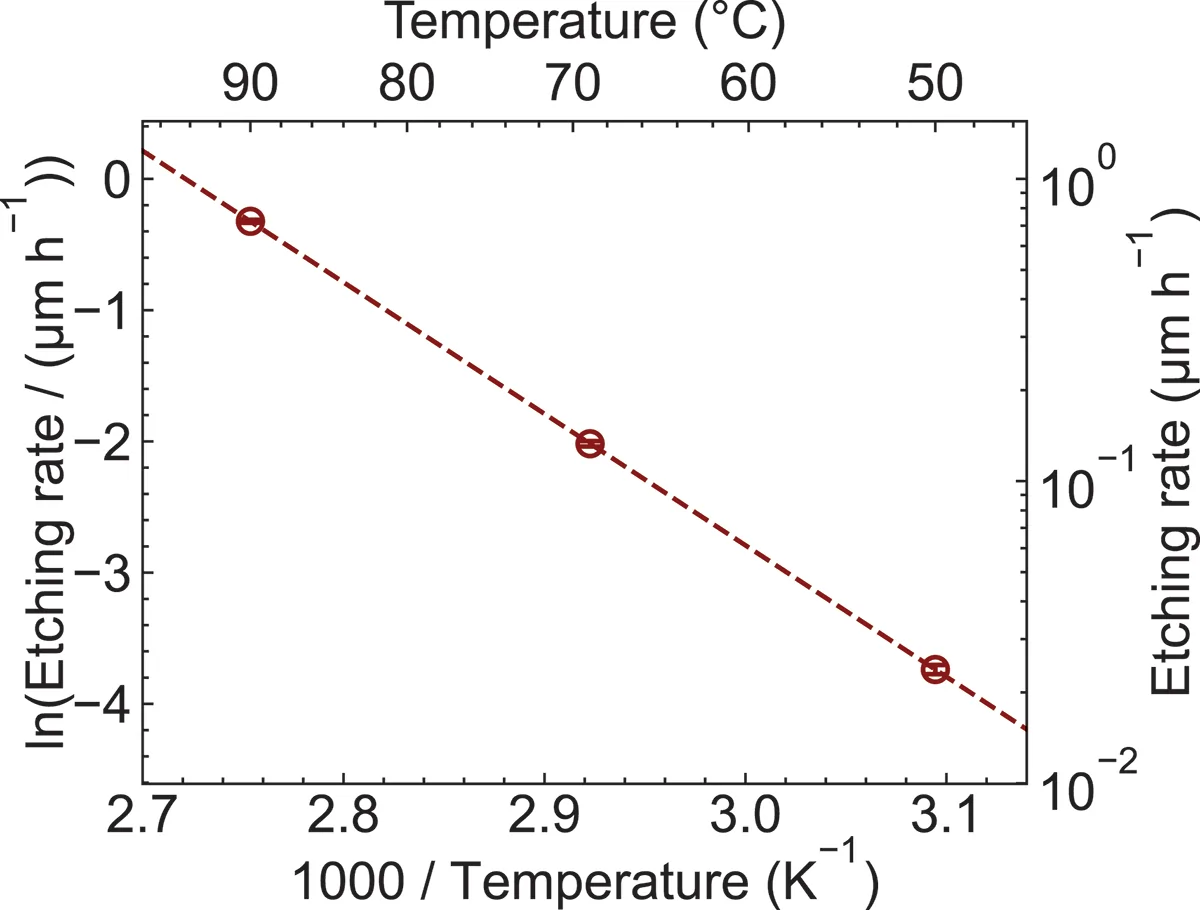
Arrhenius plot of the planar etch rate for (512) β-Ga_2_O_3_ substrates.

Before discussing anisotropic etching, we investigated the temperature dependence of the planar etch rate to clarify the wet etch kinetics. Wet etching was performed at 50 °C for 2 h, 69 °C for 1.5 h, and 90 °C for 1 h for (512) β-Ga_2_O_3_ substrates. From the depths of wet-etched depressions formed in 200 μm × 200 μm window openings, the corresponding etch rates were measured to be 0.0238 ± 0.0008, 0.133 ± 0.003, and 0.724 ± 0.009 µm h^−1^, respectively. Figure 4 shows the Arrhenius plot of the etch rates. It showed a linear relationship between the logarithm of the etch rate and reciprocal temperature, indicating surface-reaction-limited etching in this temperature range. Here, the planar etch rate can be expressed using the Arrhenius equation:

$$\mathrm{Etch}\;\mathrm{rate}\;=\;A\exp\left(-\frac{E_{a}}{\mathrm{RT}}\right),$$

where *A* is the pre-exponential factor, *E*_a_ is the activation energy, *R* is the gas constant, and *T* is the absolute temperature. From the slope of the linear fit to the Arrhenius plot, *E*_a_ was extracted to be 83.3 ± 0.1 kJ mol^−1^ (0.863 ± 0.001 eV). This value can be compared with *E*_a_ = 84.5 kJ mol^−1^ for H_3_PO_4_ etching of the (100) plane [9], *E*_a_ = 0.49 eV for H_3_PO_4_ etching of the (001) plane [11], and *E*_a_ = 83.5 kJ mol^−1^ (0.865 eV) for TMAH etching of the ($\overset{ˉ}{1}$02) plane [18].

**Figure 5.**
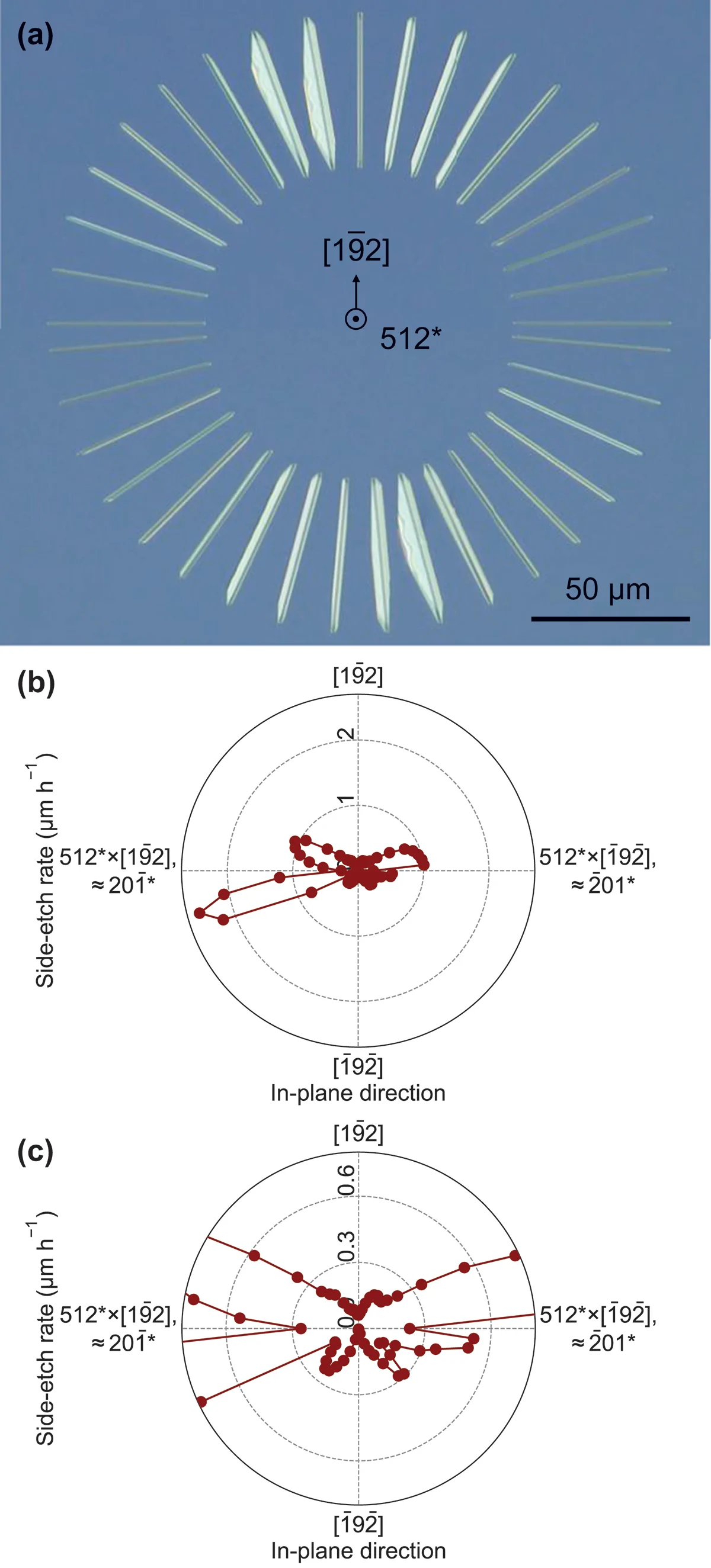
(a) Optical microscopy image of wet-etched trenches formed beneath wagon-wheel-patterned windows on a (512) β-Ga_2_O_3_ substrate. (b), (c) Polar plots of the side-etch rate derived from the wagon-wheel-patterned trenches, displayed over wide and narrow side-etch-rate ranges, respectively. Here, the symbols of ‘*’, ‘×’, and ‘≈’ have the same meanings as in the caption of Figure 1.

Next, we investigated the in-plane side-etch characteristics on the (512) β-Ga_2_O_3_ substrate, as shown in Figure 5. We used wagon-wheel-patterned linear windows with a width of 0.80 μm and a length of 50.2 μm, arranged at 10° intervals. The upper and lower halves of the windows were angularly offset by 5°, and one window in the upper half was aligned along the [1$\overset{ˉ}{9}$2] direction. TMAH etching was performed at 90 °C for 2 h. The etched trenches formed beneath the windows exhibited complicated anisotropic side-etch behavior, as observed in the optical microscopy image shown in Figure 5(a). Figures 5(b,c) show polar plots of the side-etch rates measured from the trenches in higher and lower side-etch-rate ranges, respectively. Although the polar plot has a complicated shape, it closely resembles that obtained from wagon-wheel-patterned, TMAH-etched trenches on (010) β-Ga_2_O_3_ substrates [15,17]. This similarity is consistent with the relatively small angular difference of 61.8° between the (512) and (010) planes. Based on the findings for the (010) substrate, the relatively large side-etch rates observed here are likely attributable to the high etch rates of the {$\overset{ˉ}{1}$02} planes [15,17,18]. For the trench etched along the [1$\overset{ˉ}{9}$2] direction, relatively small side-etch rates were observed near the directions normal to the {$\overset{ˉ}{2}$01} planes, suggesting the formation of {$\overset{ˉ}{2}$01} facets. Furthermore, these in-plane directions correspond to dips in the polar plots, where local minima in the side-etch rate are considered to promote the flattening of the {$\overset{ˉ}{2}$01} facets by rapidly removing slightly misoriented crystal surfaces during etching [15].

**Figure 6.**
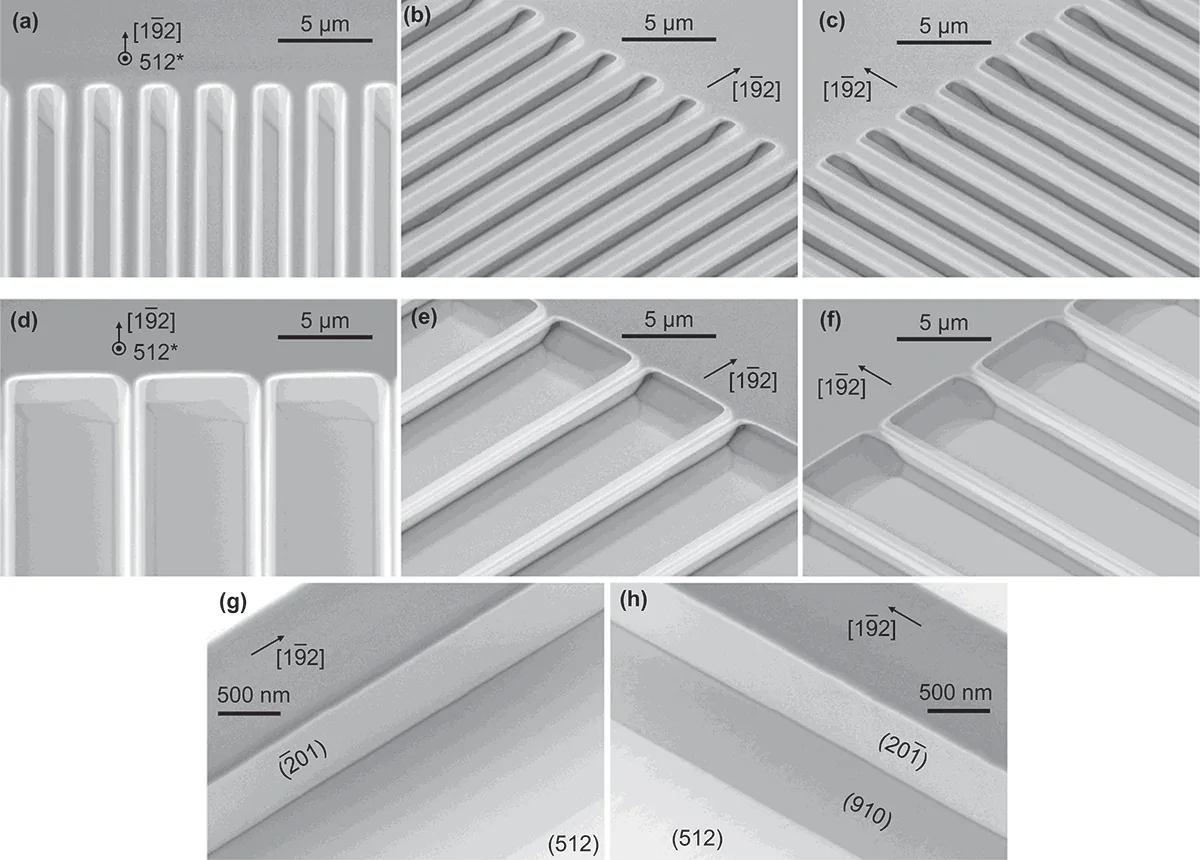
Scanning electron microscopy images of wet-etched (a)–(c) trench and (d)–(h) fin arrays aligned along the [1$\overset{ˉ}{9}$2] direction. Panels (a) and (d) are top-view images, whereas (b) and (c), (e) and (f), and (g) and (h) are 54°-tilted-view images acquired at two different stage rotation angles. Panels (a)–(f) were obtained at an acceleration voltage of 10 kV, whereas (g) and (h) were obtained at 2 kV.

**Figure 7.**
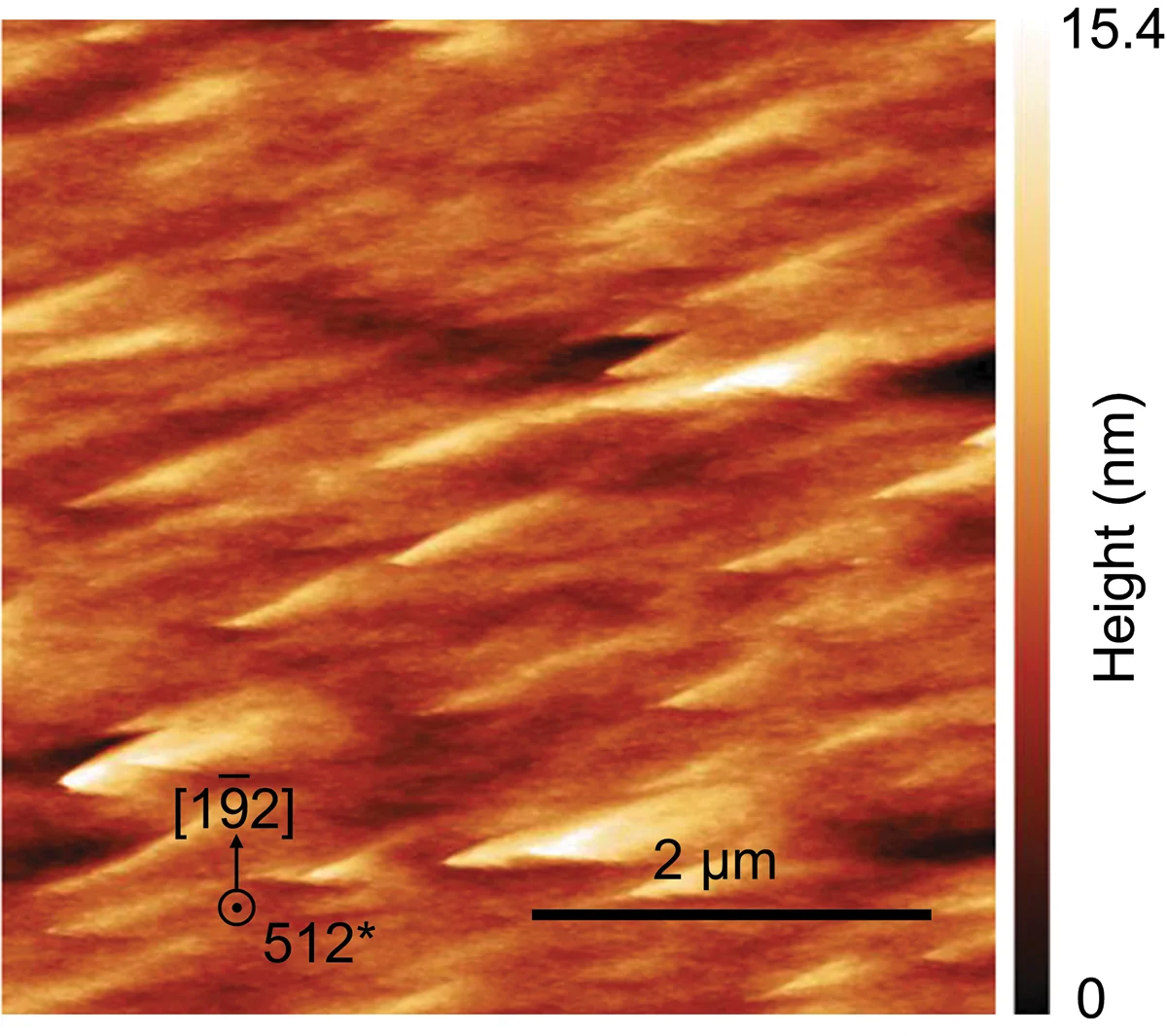
Surface morphology of the wet-etched (512) substrate observed by atomic force microscopy. The root-mean-square roughness was 1.83 nm.

Finally, we demonstrated the formation of trenches and fins by wet etching, as shown in Figure 6. Based on the discussion above, we used striped windows aligned along the [1$\overset{ˉ}{9}$2] direction, with mask and window widths of 2.13 μm and 0.87 μm, respectively, for trenches, and 1.28 μm and 5.72 μm, respectively, for fins. TMAH wet etching was carried out at 90 °C for 2 h. Trenches and fins were successfully formed in accordance with the respective mask designs, as shown in Figure 6(a–c, d–h), respectively. In both cases, the side-etched lengths were 0.44–0.56 μm, as evidenced by the SEM images acquired at an acceleration voltage of 10 kV [Figure 6(a–f)]. The sidewalls were exceptionally flat, as shown in the SEM images acquired at an acceleration voltage of 2 kV [Figure 6(g,h)]. These sidewalls were identified as {$\overset{ˉ}{2}$01} facets in the subsequent cross-sectional observations. In addition, relatively smooth, shallowly inclined (910) and unidentified facets were observed beneath the (20$\overset{ˉ}{1}$) and ($\overset{ˉ}{2}$01) facets, respectively, and formed bottom corners. The unidentified facet may comprise multiple facets or represent an intermediate stage of facet formation. In contrast, the (512) bottom surface was relatively rough. AFM observations over a 5 μm × 5 μm scan area within a 200 μm × 200 μm window opening on the same (512) substrate revealed the formation of specific anisotropic surface structures with an RMS roughness of 1.83 nm, as shown in Figure 7. Therefore, when such bottom morphology is expected to affect device characteristics, the window width should be sufficiently reduced to prevent the appearance of the (512) surface.

**Figure 8.**
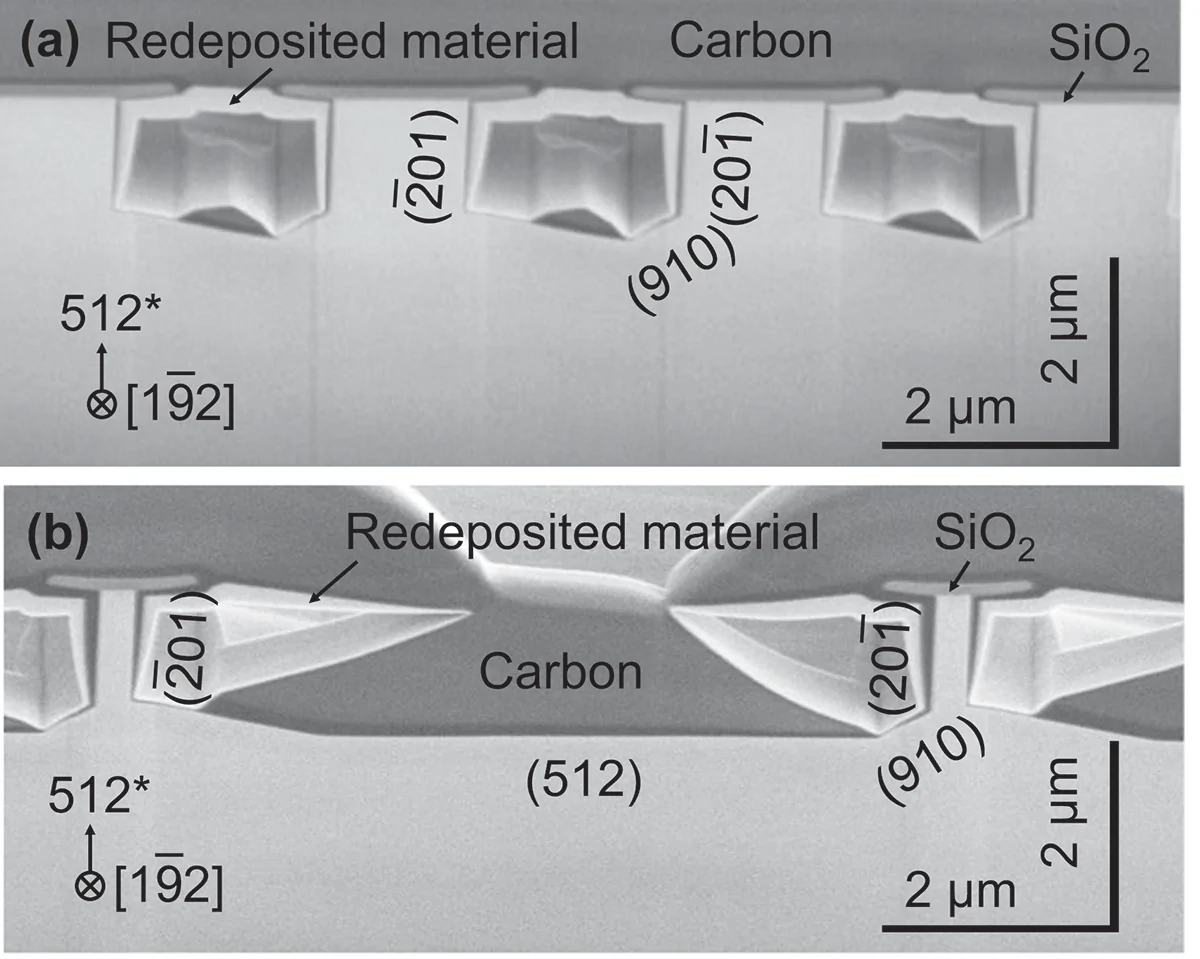
Panels (a) and (b) show 54°-tilted-view cross-sectional scanning electron microscopy images of the wet-etched trench and fin arrays, respectively. Note that the vertical and horizontal scales on the cross section are different due to the tilted view.

Cross-sectional profiles of the trenches and fins were also observed, as shown in Figure 8. The trenches were bounded by nearly vertical {$\overset{ˉ}{2}$01} facets and shallowly inclined (910) and unidentified facets [Figure 8(a)]. The (512) surface was not exposed because of the small window width. In contrast, in the fins formed with a larger window width, the (512) bottom surface appeared in addition to the facets observed in the trench structures [Figure 8(b)]. The lengths of the (910) facets were 0.52 and 0.46 μm for the structures formed with window widths of 0.87 and 5.72 μm, respectively. The corresponding lengths of the ($\overset{ˉ}{2}$01) facets were 1.17 and 0.96 μm, and those of the (20$\overset{ˉ}{1}$) facets were 1.15 and 0.91 μm. Despite the 6.6-fold difference in window width, these results indicate that the sizes of the (910) corner facets and the {$\overset{ˉ}{2}$01} near-vertical sidewall facets were comparable between the two structures. The tilt angle of the {$\overset{ˉ}{2}$01}-faceted sidewalls from the (512) surface normal was approximately 1.8°, which remains in good agreement with the crystallographically estimated angle of 1.70° when the small miscut is taken into account. Therefore, using (512) substrates with a 1.70° miscut angle could enable vertical wet etching with perfectly vertical {$\overset{ˉ}{2}$01}-faceted sidewalls. On the other hand, the inclination angle of the (910) facets from the (512) surface was approximately 31°, which also agrees well with the geometrically estimated angle of 31.1°. The process aspect ratios, defined as the (20$\overset{ˉ}{1}$) and ($\overset{ˉ}{2}$01) sidewall facet lengths divided by the corresponding side-etch lengths, were calculated to be 2.3 and 2.4 for trenches and 1.8 and 2.2 for fins, respectively. The nonnegligible difference between the two aspect ratios should be attributed to the slight tilting of the {$\overset{ˉ}{2}$01}-faceted sidewalls and the different facets formed on the bottom corners. The process aspect ratios are not sufficiently high to neglect the lateral etch component, indicating that fabricating narrow trenches is difficult, whereas fabricating narrow fins is comparatively easy.

## Summary

We demonstrated the formation of near-vertical trenches and fins with exceptionally flat {$\overset{ˉ}{2}$01}-faceted sidewalls on (512)-oriented β-Ga_2_O_3_ substrates by TMAH wet etching alone. Side-etch experiments on a ($\overset{ˉ}{2}$01) substrate indicated that {512} orientations are suitable for forming vertical structures with {$\overset{ˉ}{2}$01}-faceted sidewalls. The {$\overset{ˉ}{2}$01}-faceted sidewalls of the wet-etched trenches and fins were tilted by only approximately 1.8° from the (512) surface normal, in good agreement with the crystallographically estimated angle of 1.70°. These results indicate that the 1.70°-miscut (512)-oriented β-Ga_2_O_3_ substrate may enable vertical {$\overset{ˉ}{2}$01}-faceted sidewalls, although the influence of the miscut-induced step-and-terrace structure on the etching behavior remains to be clarified.
